# Prevalence of *Candida albicans* and non-*albicans* on the tongue dorsa of elderly people living in a post-disaster area: a cross-sectional survey

**DOI:** 10.1186/s12903-017-0342-0

**Published:** 2017-02-01

**Authors:** Toshiro Sato, Mitsuo Kishi, Miki Suda, Kiyomi Sakata, Haruki Shimoda, Hiroyuki Miura, Akira Ogawa, Seiichiro Kobayashi

**Affiliations:** 1 Division of Preventive Dentistry, Department of Oral Medicine, School of Dentistry Iwate Medical University, 1-3-27 Chuo-dori, Morioka, Iwate 020-8505 Japan; 2 Department of Hygiene and Preventive Medicine, Iwate Medical University, School of Medicine, 2-1-1 Nishitokuta, Yahaba, Iwate 028-3694 Japan; 3 Division of Dental Education, Department of Oral Medicine, School of Dentistry Iwate Medical University, 1-3-27 Chuo-dori, Morioka, Iwate 020-8505 Japan; 4 Iwate Medical University, 19-1 Uchimaru, Morioka, Iwate 020-8505 Japan

**Keywords:** *Candida albicans*, Non-*albicans*, Elderly, Post-disaster area

## Abstract

**Background:**

*Candida* species are normal commensal organisms of the mouth. However, they can cause oral mucosal and severe systemic infections in persons with reduced immune function, which is common in the very elderly. In post-disaster areas, the number of elderly residents rapidly increases due to the outflow of younger generations. Hence, we examined the prevalence of *Candida albicans* and non-*albicans* in association with oral and systemic conditions, life style, medications, and living conditions.

**Methods:**

This study was performed in 2014. Participants of this study were 266 community dwellers aged 60 years or older in Otsuchi town, which was severely damaged by the Great East Japan Earthquake and Tsunami in 2011. Oral specimens were collected from tongue dorsa by swabbing. After 48 h incubation on CHROMagar*™* medium, *C. albicans* and non-*albicans* were identified by the morphology and pigmentation of the colonies. Oral and systemic health check-ups were performed to assess the following: number of remaining teeth and periodontal status, oral hygiene, use of dentures, obesity, hypertension, hyperlipidemia, and hyperglycemia. A questionnaire addressed lifestyle, medications, and living conditions. Using the variables above, the relative factors involved in the colonization and the amounts of each type of *Candida* were determined.

**Results:**

*C. albicans* and non-*albicans* were detected in 142 (53.4%) and 63 (23.7%) participants, respectively. Multinomial logistic regression analyses revealed that the significant factors of colonization by *C. albicans* were “having decayed teeth” and “relocation from home”. Factors related to non-*albicans* colonization were “age over 80 years”, “number of remaining teeth”, “use of dentures”, and “obesity”. On the contrary, none of the parameters were related to the amount of non-a*lbicans* in the carrier, and the amount of *C. albicans* was significantly associated with “number of teeth” and “hypertension”.

**Conclusions:**

Prevalence-related factors differed between *C. albicans* and non-*albicans* colonization. In addition, other than oral status, systemic and living conditions affected the prevalence of both *C. albicans* and non-*albicans* in elderly people living in a post-disaster area.

**Electronic supplementary material:**

The online version of this article (doi:10.1186/s12903-017-0342-0) contains supplementary material, which is available to authorized users.

## Background

Over 40 species of *Candida* yeasts, the most common of which is *Candida albicans,* can cause infections in humans. In addition, *C. tropicalis, C. parapsilosis,* and *C. glabrata* are major pathogenic *Candida* species collectively referred to as non-*albicans* [1–4]. Several *Candida* species colonize mucosal surfaces in the oral cavity, digestive tract, and vagina. *Candida* are normal commensal organisms of the mouth and generally cause no problems in healthy people. In the general population, carriage rates are reported to be in the range 3 to 75% without any symptoms [5]. However, the overgrowth of *Candida* in oropharyngeal or esophageal mucosa causes a burning sensation, taste disorders, severe mucositis, or dysphagia and results in poor nutrition. From these symptoms, oral candidiasis is the most common human fungal infection, especially in the elderly or hospitalized patients [5–7]. Once the oral cavity is colonized, it becomes easier for the yeasts to reach the respiratory system, and since it is a commensal species of the gut lumen and the cutaneous surfaces, the colonization index is increasing [7, 8]. Thus, oral *Candida* poses a risk of systemic disorders as well as local mucosal infections in the elderly. Previous studies indicate that the risk factors involved in oral *Candida* colonization in the elderly include wearing dentures, poor oral/denture hygiene, and low local saliva flow [9–15]. Furthermore, elderly individuals are at risk of overgrowth of *Candida* due to several predisposing factors, such as systemic disease, decreased immune function, and the use of various medicines [7, 10, 16–19]. Therefore, the risks of *Candida* colonization should be clarified in terms of both systemic and oral conditions, especially in elderly people who are likely to harbor *Candida* and are more likely to receive hospital care.

In 2011, Japan experienced the Great East Japan Earthquake, a catastrophic disaster. Most of the severely damaged areas were rural and consisted of already aging communities compared with other areas of Japan, which has one of the largest aging populations in the world [20]. Moreover, the rate of growth of the elderly population has rapidly increased due to the outflow of the younger generation in post-disaster areas, and a considerable number of elderly people are still displaced from their homes 5 years after the disaster [21]. The extraordinary lifestyle after the disaster has had negative effects on systemic, mental, and oral health [22–24], and could affect the prevalence of oral *Candida* colonization in elderly community dwellers in the disaster areas.

The primary aim of this study was to assess the prevalence of oral *Candida* colonization and distinguish non-*albicans* from *C. albicans* in elderly community dwellers of a post-disaster area because non-*albicans* has recently been detected more frequently [25].

A second aim was to identify the factors associated with oral *Candida* colonization among demographic, oral, and systemic conditions, lifestyle, medication status, and living conditions.

## Methods

### Study design and population

**Fig. 1:**
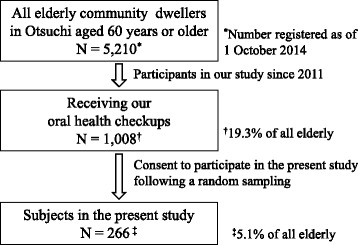
Sample selection and description of the study population

This was a cross-sectional study involving clinical surveys, a questionnaire, and microbiological examinations using culture methods. The participants were community dwellers aged 60 years or over in Otsuchi, located on the Pacific Coast of Iwate Prefecture, which experienced some of the most severe damage from the Great East Japan Earthquake and Tsunami. However, the area suffered no effects of radiation from the Fukushima Daiichi Nuclear Disaster because the distance from the power station was over 200 km. In 2014, from individuals who attended an annual oral health checkup that we had carried out since 2011, 266 candidates aged 60 years or older who lived in the tsunami inundated area at the disaster site, were randomly recruited to an additional examination to detect *Candida* species (Fig. 1). Detailed methods of initial subject recruitment were reported in our previous paper [24]. All the candidates gave informed consent to participate in this study after receiving sufficient information. The mean age and standard deviation of our participants were 72.3 and 7.0. None of the participants used antibiotics, or antifungal medications during the survey.

The study protocol was approved by the Medical Ethics Committee of Iwate Medical University School of Medicine (H23-69) and School of Dentistry (01214).

### Sample collection and identification of *Candida* species

Oral samples were collected by swabbing 10 times vertically from the circumvallate papillae to the tip of the tongue with a sterile cotton swab. Each swab was immediately immersed into 2 mL of sterile phosphate buffered saline (PBS, pH 7.4) in a stock tube and stored on ice until inoculation. After stirring with a Vortex^®^ Mixer, the cotton swab was removed from the PBS and the suspension was inoculated onto a CHROMagar Candida^TM^ plate (CHROMagar Microbiology, Paris, France) within 8 h of collection, because we confirmed in our preliminary study that the time elapsed would not affect the culture results. After 48 h of incubation, all colonies observed on CHROMagar™ were identified by their morphology and pigmentation according to the manufacturer’s instructions and a previous study [26]. In addition, the colony forming unit/mL (CFU) was calculated for both *C. albicans* and non-*albicans*.

### Oral examinations

Oral examinations were performed by a skilled dentist. Dental caries status was assessed according to the World Health Organization (WHO) method [27] with some modifications as follows: (1) a tooth with treated or untreated root caries was recorded as a filled or decayed, and (2) a remaining root without a crown was counted as a present tooth. Periodontal conditions were assessed using the Community Periodontal Index (CPI) procedures and diagnostic criteria, which are also recommended by the WHO. In addition, oral hygiene status was assessed as one of three grades: poor, fair, or good. Information on denture use was obtained from both oral examination and interview. In later analyses, participants with a CPI code of 3 or 4 (having periodontal pockets) were summarized, and oral hygiene status was categorized as poor or other status.

### Systemic data

Body mass index (BMI, kg/m^2^), blood pressure (BP, mmHg) and biochemical data, including high-density lipoprotein cholesterol (HDLC; mg/dL), low-density lipoprotein cholesterol (LDLC; mg/dL), and glycated hemoglobin (HbA1c, %) were obtained from the results of health check-ups that the municipality supplied as a public health service. The health check-up was performed according to a Japanese national guideline (the Specific Health Checkups) [28]. Participants were placed in one of two groups depending on the measurements, using the following cutoff values: BMI ≥25 (obesity), systolic BP ≥ 140 or diastolic BP ≥90 (hypertension), HDLC ≤ 34 or LDLC ≥140 (dyslipidemia), and HbA1c ≥5.6% (hyperglycemia).

### Self-reported data

Self-report questionnaires were administrated to assess lifestyle (smoking and drinking status), daily medications (antihypertension drugs, diabetes drugs, hypnotics), and whether the participants’ current accommodation was the same as that before the disaster (relocation from home). The choices of answers for smoking status were current smoker, past smoker, and non-smoker. We categorized those choices into current smoker or other in our statistical analyses. Similarly, choices for drinking status (daily drinker, occasional drinker, and no drinker) were summarized into daily drinker or other.

### Statistical analyses

The prevalence of *Candida* was examined in all 266 participants. In subsequent factor analyses, two participants were excluded because certain data was missing. To determine the factors related with colonization of *C. albicans* or non-*albicans*, we assessed risk measurements by multinomial logistic regression models followed by crude analyses using Chi-squared tests. To compare the amounts of *Candida* species between the parameters, the Mann-Whitney *U* test or Kruskal-Wallis test was used, since the distribution of *Candida* amounts were non-normal after logarithmic transformation. Post hoc multiple comparisons after the Kruskal-Wallis test were performed using the Mann-Whitney *U* test with Bonferroni correction. To reveal the relationship between number of remaining teeth and log CFUs of *C. albicans* and non-*albicans*, Spearman’s rank correlation analysis and logistic regression analysis using a cutoff value (log CFU = 1.5) were performed. A two-sided *p-*value of less than 0.05 was considered statistically significant. All statistical analyses were conducted using the software program SPSS version 24.0 for Windows (IBM).

## Results

### Prevalence of *C. albicans* and non-*albicans*

*Candida* species in tongue dorsa were detected in 162 participants (60.9%). *C. albicans* was the most frequently detected in (*n* = 142; 53.4%) participants, followed by *C. glabrata* (*n* = 60; 22.6%), *C. tropicalis* (*n* = 5; 1.8%), and *C. parapsilosis* (*n* = 2; 0.7%). *C. krusei* were not detected in any of our participants. At least one non-*albicans* species were detected in 63 (23.7%) of the participants (Table 1). In addition, 61.1% and 11.7% of *Candida* carriers exclusively harbored either *C. albicans* or *C. glabrata*, respectively. A combination of *C. albican*s and *C. glabrata* was found in 23.5% of *Candida* carriers, and other combinations were found in only 3.7%.

**Table 1:** Detection rates of *Candida* in elderly community dwellers (*n* = 266) in a post-disaster area

*Candida* spp.	Number of carriers	% of entire subjects	% of carriers of any *Candida*
*C. albicans*	142	53.4	87.7
*C. glabrata*	60	22.6	37.0
*C. tropicalis*	5	1.8	3.1
*C. parapsilosis*	2	0.7	1.2
*C. krusei*	0	0	0
Any non-*albicans*	63	23.7	38.9
Any *Candida* spp.	162	60.9	-

### Factors associated with colonization by *C. albicans* and non-*albicans*

Tables 2 and 3 show the significant factors related to colonization by *C. albicans* and non-*albicans* in the demographic data and the parameters measured in this study (see Additional file: 1 for complete data). A crude odds ratio (COR) with a 95% confidence interval (CI) was calculated from a bi-variable Chi-squared test, and the adjusted odds ratio (AOR) with 95% CI was calculated from multinomial logistic regression analysis in which the variables were all adjusted for each other. In the bi-variable models, participants “having one or more decayed teeth”, “poor oral hygiene” and “relocation from home” harbored *C. albicans* at a significantly higher frequency (COR = 3.35; 95% CI = 1.70–6.63, COR = 2.42; 95% CI = 1.18–4.99 and COR = 1.45; 95% CI = 1.06–1.99, respectively). For colonization of non-*albicans*, “over 80 years old”, “less than 20 remaining teeth/edentulous”, “use of dentures” and “obesity” were significant factors associated with colonization (COR = 2.21; 95% CI = 1.36–5.34, COR = 1.84/2.39; 95% CI = 1.51–2.42/1.83–3.12, COR = 1.56; 95% CI = 1.37–1.77 and COR = 1.45; 95% CI = 1.06–2.00, respectively). In the multivariable models, “having one or more decayed teeth” and “relocation from home” were significant factors for colonization by *C. albicans* similarly to bi-variable analyses, although “poor oral hygiene” was not significant (AOR = 3.51; 95% CI = 1.60–7.67, AOR = 2.17; 95% CI = 1.25–3.78 and AOR = 2.01; 95% CI = 0.77–5.24, respectively). The same factors were significant to colonization by non-albicans in bi-variable analyses, excluding “less than 20 teeth” (AOR = 3.37; 95% CI = 0.88–12.9). In addition, the odds ratios of “over 80 years old”, “edentulous”, “use of dentures” and “obesity” were raised after adjustment for each other (AOR = 2.58; 95% CI = 1.12–5.71, AOR = 5.99; 95% CI = 1.37–26.3, AOR = 4.02; 95% CI = 1.05–15.4 and AOR = 2.25; 95% CI = 1.15–4.40, respectively).

**Table 2:** Significant factors related to colonization of *C. albicans* in surveyed parameters in this study (*N* = 264)

Parameter	*n*	COR (95% CI) *p*-value	AOR (95% CI) *p*-value	Number of positive (%)
Having one or more decayed teeth				
Yes	53	3.35 (1.70–6.63) <0.01	3.51 (1.60–7.67) <0.01	40 (75.5)
No^a^	211	1.00	1.00	101 (47.9)
Oral hygiene				
Poor	34	2.42 (1.18–4.99) <0.01	2.01 (0.77–5.24) 0.15	25 (73.5)
Fair/Good	230		1.00	116 (50.4)
Relocation from home				
Yes	104	1.45 (1.06–1.99) 0.02	2.17 (1.25–3.78) <0.01	65 (62.5)
No	160	1.00	1.00	76 (47.5)

Parameters include significant factors in either bivariate or multivariate analysis, *COR* crude odds ratio from bivariate analysis, *AOR* adjusted odds ratio from multinomial logistic regression analysis; *CI* confidence interval

^a^The reference categories are in the last row for all explanatory variables

**Table 3:** Significant factors related to colonization of Non-a*lbicans* in surveyed parameters in this study (*N* = 264)

	*n*	COR (95% CI) *p*-value	AOR (95% CI) *p*-value	Number of positive (%)
Age (in years)				
≥ 80	44	2.21 (1.36–5.34) <0.01	2.58 (1.12–5.71) 0.02	18 (40.9)
< 80^a^	220	1.00	1.00	45 (20.5)
Number of remaining teeth				
Edentulous	73	2.39 (1.83–3.12) <0.01	5.99 (1.37–26.3) 0.02	27 (37.0)
1-19	107	1.84 (1.51–2.42) <0.01	3.37 (0.88–12.9) 0.08	32 (29.9)
20 or more	84	1.00	1.00	4 (4.8)
Use of dentures				
Yes	180	1.56 (1.37–1.77) <0.01	4.02 (1.05–15.4) 0.04	59 (32.8)
No	84	1.00	1.00	4 (4.8)
Obesity (BMI ≥ 25)				
Yes	99	1.45 (1.06–2.00) 0.04	2.25 (1.15–4.40) 0.02	31 (31.3)
No	165	1.00	1.00	32 (19.4)

Parameters include significant factors in either bivariate or multivariate analysis, *COR* crude odds ratio from bivariate analysis, *AOR* adjusted odds ratio from multinomial logistic regression analysis; *CI* confidence interval

^a^The reference categories are in the last row for all explanatory variables

### Factors related to the amounts of *C. albicans* and non-*albicans* among the carriers

We compared the amounts (log CFU) of *Candida* between same parameters as the examination for colonization-related factors of each *Candida* carrier. Larger amounts of *C. albicans* were detected in edentulous subjects than in dentate subjects among *C. albicans* carriers. In addition, participants with hypertension and a daily drinking habit harbored greater amounts of *C. albicans*. However, the amounts of non-*albicans* among the carriers were no different between any of the categories (Table 4).

**Table 4:** Comparisons of colony amounts of *Candida* species by demographic characteristics, oral conditions, systemic conditions, lifestyle, medications, and relocation from home in carriers

	*C. albicans*			Non-*albicans*		
	No. of carriers	Mean log CFU (SD)	*p*-value^a^	No. of carriers	Mean log CFU (SD)	*p*-value
Sex						
Female	78	1.44 (0.65)	0.72	42	1.91 (0.92)	0.63
Male^a^	63	1.44 (0.66)		21	1.73 (0.74)	
Age (in years)						
≥ 80	29	1.59 (0.74)	0.24	18	1.87 (0.88)	0.84
< 80	112	1.40 (0.63)		45	1.85 (0.86)	
Having one or more decayed teeth						
Yes	40	1.45 (0.60)	0.77	13	1.56 (0.62)	0.31
No	101	1.43 (0.68)		50	1.93 (0.90)	
Number of remaining teeth						
Edentulous	33	1.88 (0.67)	<0.01^b^	27	2.17 (0.93)	0.09
1-19	60	1.35 (0.61)		32	1.62 (0.78)	
20 or more	48	1.25 (0.56)		4	1.55 (0.24)	
Having periodontal pockets						
Yes	41	1.32 (0.66)	0.16	14	1.69 (0.71)	0.55
No	100	1.49 (0.65)		49	1.90 (0.60)	
Oral hygiene						
Poor	25	1.39 (0.65)	0.70	8	1.69 (0.73)	0.75
Fair/Good	116	1.45 (0.66)		55	1.88 (0.88)	
Use of denture						
Yes	93	1.52 (0.68)	0.07	59	1.88 (0.88)	0.49
No	48	1.29 (0.58)		4	1.45 (0.40)	
Obesity (BMI ≥ 25)						
Yes	11	1.42 (0.72)	0.86	31	1.93 (0.89)	0.50
No	130	1.44 (0.65)		32	1.78 (0.84)	
Hypertension (systolic BP ≥ 140 or diastolic BP ≥90)						
Yes	38	1.61 (0.63)	0.04	24	1.84 (0.77)	0.96
No	103	1.37 (0.65)		39	1.86 (0.92)	
Dyslipidemia (HDLC ≤ 34 or LDLC ≥140)						
Yes	37	1.31 (0.57)	0.20	14	1.90 (0.92)	0.99
No	104	1.48 (0.68)		49	1.84 (0.85)	
Hyperglycemia (HbA1c ≥5.6)						
Yes	31	1.58 (0.67)	0.14	17	2.07 (0.71)	0.50
No	110	1.40 (0.65)		46	1.77 (0.90)	
Current smoker						
Yes	11	1.42 (0.72)	0.86	5	1.63 (0.88)	0.58
No	130	1.44 (0.65)		58	1.87 (0.86)	
Daily drinker						
Yes	19	1.72 (0.67)	0.40	7	1.81 (1.26)	0.54
No	122	1.39 (0.64)		56	1.86 (0.81)	
Taking antihypertensive drugs						
Yes	83	1.48 (0.65)	0.30	38	1.98 (0.83)	0.09
No	58	1.37 (0.66)		25	1.65 (0.88)	
Taking diabetes drugs						
Yes	8	1.45 (0.60)	0.80	5	2.11 (0.82)	0.41
No	133	1.43 (0.66)		58	1.83 (0.87)	
Taking hypnotics						
Yes	25	1.49 (0.76)	0.81	12	2.15 (0.79)	0.14
No	116	1.42 (0.63)		51	1.78 (0.87)	
Relocation from home						
Yes	65	1.40 (0.65)	0.50	25	1.82 (0.84)	0.72
No	76	1.47 (0.66)		38	1.88 (0.89)	

^a^Statistical differences were examined by Mann-Whitney *U* test for bivariate combinations, and Kruskal-Wallis test for comparison of 3 groups

^b^Post-hoc multiple comparisons using Bonferroni adjustment indicated that there was statistical significance between edentulous and others

### Correlation between amounts of *C. albicans* and non-*albicans* with number of remaining teeth in denture wearers

Spearman’s correlation coefficient between amounts (log CFU) of *Candida*, number of remaining teeth and age were examined in each *Candida* carrier wearing a denture (Table 5). There were significant correlations between log CFU and number of remaining teeth in both groups of carriers (ρ = –0.40, *p*-value < 0.01 for *C. albicans*; ρ = –0.38, *p*-value < 0.01 for non-*albicans*), although we also found significant relationships between age and number of remaining teeth (ρ = –0.34, *p*-value < 0.01 for *C. albicans*; ρ = –0.40, *p*-value < 0.01 for non-*albicans*). To adjust for possible confounders, we applied logistic regression models using a cutoff value. In these models, the dependent variable was a high amount of each type of *Candida* (CFU ≥ 1.5) and the independent variables were number of remaining teeth and age in years. As Table 6 shows, after a stepwise procedure, the number of remaining teeth was selected as a significant factor of high amounts of both *C. albicans* and non-*albicans* (adjusted partial regression coefficient = 0.94; 95% CI = 0.88–0.99 and adjusted partial regression coefficient = 0.91; 95% CI = 0.83–0.98).

**Table 5:** Relationships between colony amounts of *Candida* species, age, and number of remaining teeth in carriers wearing dentures

	*C. albicans* (*n*=93)			Non-*albicans* (*n*=59)		
	Log CFU	Number of remaining teeth	Age	Log CFU	Number of remaining teeth	Age
Log CFU		–0.40 (<0.01)	–0.18 (0.09)		–0.38 (<0.01)	0.10 (0.45)
Number of remaining teeth	–0.40 (<0.01)		–0.34 (<0.01)	–0.38 (<0.01)		–0.40 (<0.01)
Age	–0.18 (0.09)	–0.34 (<0.01)		0.10 (0.45)	–0.40 (<0.01)	

Value shows Spearman’s correlation coefficient (*p*-value)

**Table 6:** Relationship between age, number of remaining teeth, and colony amounts of *Candida* by logistic regression models^a^

*C. albicans* (*n* = 93)					
	Variable	Crude partial regression coefficient	Adjusted partial regression coefficient	95% CI	*p*-value
First step	Number of remaining teeth	-0.06	0.94	0.53–0.94	0.053
	Age	0.01	1.02	0.95–1.08	0.65
Second (final) step	Number of remaining teeth	-0.07	0.94	0.88–0.99	0.03
Non- albicans (*n* = 59)					
	Variable	Partial regression coefficient	Adjusted partial regression coefficient	95% CI	*p*-value
First step	Number of remaining teeth	-0.11	0.90	0.82–0.98	0.02
	Age	-0.02	0.62	0.90–1.06	0.62
Second (final) step	Number of remaining teeth	-0.10	0.91	0.83–0.98	0.02

^a^Dependent variable: log CFU ≥ 1.5; 1, log CFU < 1.5; 0

## Discussion

In this study, the subjects were community dwellers in a post-disaster area. There have been several reports on prevalence of *Candida* species*,* mainly in institutionalized elderly, or in patients with HIV, carcinoma, or diabetes [9, 29–34]. However, information on the carriage rates of oral *Candida* in community dwellers is limited. Wang et al. [18] reported that 68.6% of community-dwelling elderly Japanese people aged 75 years were positive for total *Candida*. In addition, Goto et al. [35] reported prevalence rates of 63.6% for total *Candida*, and 53.4% for *C. albicans* in an elderly population aged 65–74. These rates are very close to those of our subjects (60.9% for total *Candida* and 53.4% for *C. albicans*); however, *Candida* prevalence varies widely in the oral cavities of healthy individuals according to several previous studies [5, 25, 36]. The factors associated with *Candida* colonization in the oral cavities of elderly people include age, use of dentures, poor oral/denture hygiene, and low saliva flow, in addition to systemic diseases [9–15]. The similar overall carriage rates of *Candida* found in these studies and ours in similarly aged Japanese elderly people may indicate that differences among oral or systemic conditions do not affect the colonization status of *Candida* even in post-disaster areas.

We found differences among the factors associated with oral colonization between *C. albicans* and non-*albicans* (Tables 2 and 3). The oral factors affecting colonization presented in previous studies, such as use of dentures or number of remaining teeth, were associated exclusively with non-*albicans* in our subjects. On the contrary, quantitative analysis revealed that none of the parameters were associated with the colony counts of non-*albicans,* although edentulous subjects harbored significantly greater amounts of *C. albicans*. In addition, denture users tended to have a greater amount of *C. albicans* although this was not statistically significant (Table 4). The dissimilarities between *Candida* species both in our qualitative and quantitative analyses suggested differences in the time of colonization by *C. albicans* and non-*albicans*. Elderly subjects might harbor stable non-progressive colonization by *C albicans*, while non- *albicans* colonization could be progressive. Therefore, despite the number of remaining teeth, or use of dentures being common factors in the prevalence of both *Candida* species, they might have largely affected colonization by non-*albicans* and growth of *C. albicans,* at least in our subjects. On the other hand, colonization by *C. albicans* related significantly to “having one or more decayed teeth”. The relationship between *Candida* carriage and dental caries status has mainly been reported in children [37–39]. In these studies, *C. albicans* or total *Candida* carriage was generally associated with the prevalence of dental caries. However, race, age, country, and the oral status of the subjects in these previous studies were very different from our study, which makes it difficult for us to compare our results. In addition, the previous studies aimed to clarify the etiology of *Candida* in dental caries. Rather, we presumed that “having decayed teeth” was a consequence of poor living conditions after the disaster. Affected individuals were exempt from medical insurance premiums and payments at medical institution counters, including dental facilities, by the Japanese government and the local municipalities [40]. However, despite the existence of these services, subjects with decayed teeth did not appear to have received dental care for some reason, perhaps due to lack of transportation, social networks, and so on. *C. albicans* was also found more frequently in subjects living in a different house from their own home (many of them were in temporary housing). Moreover, significant trends in the prevalence of *Candida* species were also found in general health conditions such as obesity and colonization by non-*albicans,* and hypertension and colony counts of *C. albicans* in *C. albicans* carriers. The reasons are unclear, however, such symptoms tend to originate from an unhealthy lifestyle. Therefore, our findings suggested that living conditions and systemic disorders derived from lifestyle other than oral factors could affect the prevalence of *Candida* elderly people living in post-disaster areas.

Analyses of denture wearers harboring *C. albicans* or non-*albicans* revealed negative correlations between number of remaining teeth and the colony counts of both *C. albicans* and non-*albicans* after adjusting for the confounders number of remaining teeth and age (Table 5 and 6). Thus, positive correlations were found between the number of missing teeth and the quantities of *Candida*. Accordingly, dentures covering large areas of oral mucosa could be a risk factor for high amounts of both *C. albicans* and non-*albicans*. Although studies on the relationship between dentures and the amount of *Candida*, or denture size and frequency of *Candida* colonization have occasionally been reported [41, 42], our research might be the first study on denture size and the amount of *Candida,* especially in subjects without symptoms. However, it is unclear whether the colony counts of *Candida* is a risk factor for the incidence of candidiasis. Further investigation into the significance of amounts of the colonization of *Candida* is needed.

This study had several limitations. First, the number of subjects represented a small proportion of the entire community (population of 60 years or older was 5,210 on 1 October 2014, according to a government report). However, our subjects were probability samples recruited from all participants who took part in our dental health check-ups. We believe they represented all residents to a reliable degree because the total number of participants (*n* = 1,008, Fig. 1) were sufficient to represent the entire elderly population. Second, we did not assess dryness of the mouth since this could not be conducted in our field survey. Based on the previous studies, this factor is frequently associated with *Candida* prevalence. Our models accounting for the prevalence of *Candida* would have been more accurate if this factor had been included. Third, being a cross-sectional study, it essentially lacked data from the affected region before the disaster, although we expect to provide supplemental data in a follow-up study. Finally, whether our findings are specific to post-disaster areas remains unclear given the lack of comparative data from non-disaster situation. This study was conducted as part of a large-scale cohort study aimed at supporting victims of the Great East Japan Earthquake. Since the research design did not include control areas at the beginning of the study in 2011 [22–24], it was not possible to establish control areas during the present survey because of time and budget constraints. However, compared to some previous studies for similarly aged elderly people living in ordinary conditions, our results partially identified relative post-disaster risks for the prevalence of *Candida* and can be generalized to some degree.

## Conclusions

In our participants, the factors related to colonization or amounts differed between *C. albicans* and non-*albicans*. It is suggested that there is a difference in the time of colonization between *Candida* species. In addition, the amounts of both *Candida* types were related to greater numbers of missing teeth. Perhaps the amount of *Candida* on the tongue dorsa increased with increases in size of the area covered by the denture. Further, systemic and living conditions, other than oral status, could affect the prevalence of both *C. albicans* and non-*albicans* among elderly people living in a post-disaster area.

## Additional file

Additional file 1: Relationships of demographic character, oral conditions, systemic conditions, lifestyle, medications and relocation from home with colonization of *C. albicans* and non-*albicans* (*N* = 264). Results of multinomial logistic regression analysis for colonization of *C. albicans* and non-*albicans*. Relationships of *C. albicans* and non-*albicans* colonization with all independent variables examined in this study are presented in this table. (DOCX 22 kb)

## Abbreviations

AOR: Adjusted odds ratio
BMI: Body mass index
BP: Blood pressure
CFU: Colony forming unit/mL
CI: Confidential interval
COR: Crude odds ratio
CPI: Community Periodontal Index
HbA1c: Glycosylated hemoglobin
HDLC: High-density lipoprotein cholesterol
LDLC: Low-density lipoprotein cholesterol
SD: Standard deviation
WHO: World Health Organization
