# Supplementary material for: Prevalence of Candida albicans and non-albicans on the tongue dorsa of elderly people living in a post-disaster area: a cross-sectional survey
Source: BMC Oral Health. 2017 Feb 1;17:51. doi: 10.1186/s12903-017-0342-0 (PMC5288978; doi:10.1186/s12903-017-0342-0)
Supplement: Additional file 1: — Relationships of demographic character, oral conditions, systemic conditions, lifestyle, medications and relocation from home with colonization of C. albicans and non-albicans (N = 264). Results of multinomial logistic regression analysis for colonization of C. albicans and non-albicans. Relationships of C. albicans and non-albicans colonization with all independent variables examined in this study are presented in this table. (DOCX 22 kb) [file 12903_2017_342_MOESM1_ESM.docx]

**Relationships of demographic character, oral conditions, systemic conditions, lifestyle, medications and relocation from home with colonization of *C. albicans* and non-*albicans* (N=264).**

|  | **Colonization of *Candida*** | | | | | | | |
| --- | --- | --- | --- | --- | --- | --- | --- | --- |
|  | ***C. albicans*** | | | |  | **Non-*albicans*** | | |
|  | ***n*** | **COR (95% CI)**  ***p*-value** | **AOR (95% CI)**  ***p*-value** | **Number of positive (%)** |  | **COR (95% CI)**  ***p*-value** | **AOR (95% CI)**  ***p*-value** | **Number of positive (%)** |
| **Sex** |  |  |  |  |  |  |  |  |
| Women | 150 | 0.945 (0.77-1.12)  0.62 | 0.83 (0.43-1.58)  0.56 | 78 (52.0) |  | 1.24 (0.99-154)  0.08 | 1.69 (0.75-3.79)  0.20 | 42 (28.0) |
| Men^a^ | 114 | 1.00 | 1.00 | 63 (55.3) |  | 1.00 | 1.00 | 21 (18.4) |
| **Age (in years)** |  |  |  |  |  |  |  |  |
| ≥ 80 | 44 | 1.69 (0.95-3.00)  0.07 | 2.12 (0.99-4.56)  0.054 | 29 (65.9) |  | 2.21 (1.36-5.34)  <0.01 | 2.58 (1.12-5.71)  0.02 | 18 (40.9) |
| < 80 | 220 | 1.00 | 1.00 | 112 (50.9) |  | 1.00 | 1.00 | 45 (20.5) |
| **Having one or more decayed teeth** | | |  |  |  |  |  |  |
| Yes | 53 | 3.35 (1.70-6.63)  <0.01 | 3.51 (1.60-7.67)  <0.01 | 40 (75.5) |  | 1.04 (0.59-1.81)  0.86 | 1.40 (0.57-3.44)  0.46 | 13 (24.5) |
| No | 211 | 1.00 | 1.00 | 101 (47.9) |  | 1.00 | 1.00 | 50 (23.7) |
| **Number of present teeth** | | | |  |  |  |  |  |
| Edentulous | 73 | 0.77 (0.55-1.09)  0.15 | 0.77 (0.27-2.15)  0.61 | 33 (45.2) |  | 2.39 (1.83-3.12)  <0.01 | 5.99 (1.37-26.3)  0.02 | 27 (37.0) |
| 1-19 | 107 | 0.98 (0.76-1.26)  1.00 | 0.81 (0.33-1.99)  0.64 | 60 (56.1) |  | 1.84 (1.51-2.42)  <0.01 | 3.37 (0.88-12.9)  0.08 | 32 (29.9) |
| 20 or more | 84 | 1.00 | 1.00 | 48 (57.1) |  | 1.00 | 1.00 | 4 (4.8) |
| **Having periodontal pockets** | | |  |  |  |  |  |  |
| Yes | 72 | 1.15 (0.77-1.72)  0.49 | 0.90 (0.45-1.79)  0.76 | 41 (56.9) |  | 0.77 (0.46-1.28)  0.34 | 1.16 (0.47-1.87)  0.96 | 14 (19.4) |
| No | 192 | 1.00 | 1.00 | 100 (52.1) |  | 1.00 | 1.00 | 49 (25.5) |
| **Oral hygiene** | |  |  |  |  |  |  |  |
| Poor | 34 | 2.42 (1.18-4.99)  < 0.01 | 2.01 (0.77-5.24)  0.15 | 25 (73.5) |  | 0.98 (0.47-2.06)  1.00 | 1.86 (0.60-5.75)  0.28 | 8 (23.5) |
| Fair/Good | 230 |  | 1.00 | 116 (50.4) |  | 1.00 | 1.00 | 55 (23.9) |
| **Use of denture** | | |  |  |  |  |  |  |
| Yes | 180 | 0.93 (0.79-1.10)  0.43 | 1.04 (0.43-2.49)  0.93 | 93 (51.2) |  | 1.56 (1.37-1.77)  <0.01 | 4.02 (1.05-15.4)  0.04 | 59 (32.8) |
| No | 84 | 1.00 | 1.00 | 48 (57.1) |  | 1.00 | 1.00 | 4 (4.8) |
| **Obesity** (BMI≥25) |  |  |  |  |  |  |  |  |
| Yes | 99 | 0.93 (0.68-1.27)  0.70 | 0.75 (0.43-1.31)  0.31 | 51 (51.5) |  | 1.45 (1.06-2.00)  0.04 | 2.25 (1.15-4.40)  0.02 | 31 (31.3) |
| No | 165 | 1.00 | 1.00 | 90 (54.5) |  | 1.00 | 1.00 | 32 (19.4) |
| **Hypertension** (systolic BP ≥ 140 or diastolic BP ≥90) | | | |  |  |  |  |  |
| Yes | 85 | 0.71 (0.50-1.00)  0.06 | 0.57 (0.32-1.01)  0.053 | 38 (44.7) |  | 1.26 (0.86-1.83)  0.28 | 1.25 (0.64-2.42)  0.52 | 24 (28.2) |
| No | 179 | 1.00 | 1.00 | 103 (57.5) |  | 1.00 | 1.00 | 39 (21.8) |
| **Dyslipidemia** (HDLC ≤ 34 or LDLC ≥140) | | | |  |  |  |  |  |
| Yes | 67 | 1.08 (0.71-1.63) | 1.19 (0.64-2.24)  0.58 | 37 (55.2) |  | 0.84 (0.50-1.41)  0.62 | 0.93 (0.43-2.06)  0.87 | 14 (20.9) |
| No | 197 | 1.00 | 1.00 | 104 (52.8) |  | 1.00 | 1.00 | 49 (24.9) |
| **Hyperglycemia** (HbA1c ≥5.6) | | |  |  |  |  |  |  |
| Yes | 59 | 0.97 (0.62-1.52)  0.88 | 0.88 (0.43-1.81)  0.74 | 31 (52.5) |  | 1.29 (0.79-2.10)  0.30 | 1.50 (0.63-3.61)  0.36 | 17 (28.8) |
| No | 205 | 1.00 | 1.00 | 110 (53.7) |  | 1.00 | 1.00 | 46 (22.4) |
| **Current smoker** | | | |  |  |  |  |  |
| Yes | 20 | 1.07 (0.46-2.49)  1.00 | 1.02 (0.33-3.10)  0.98 | 11 (55.0) |  | 1.06 (0.40-2.81)  1.00 | 2.16 (0.59-7.82)  0.24 | 5 (25.0) |
| No | 244 | 1.00 | 1.00 | 130 (53.3) |  | 1.00 | 1.00 | 58 (23.8) |
| **Daily drinker** | |  |  |  |  |  |  |  |
| Yes | 44 | 0.66 (0.38-1.14)  0.14 | 0.60 (0.26-1.39)  0.24 | 19 (43.2) |  | 0.60 (0.28-1.29)  0.24 | 0.57 (0.20-1.64)  0.30 | 7 (15.9) |
| No | 220 | 1.00 | 1.00 | 122 (55.5) |  | 1.00 | 1.00 | 56 (25.5) |
| **Taking antihypertension drugs** | | |  |  |  |  |  |  |
| Yes | 145 | 1.17 (0.93-1.46)  0.18 | 1.52 (0.86-2.69)  0.15 | 83 (57.2) |  | 1.13 (0.89-1.44)  0.38 | 0.93 (0.47-1.82)  0.83 | 38 (26.2) |
| No | 119 | 1.00 | 1.00 | 58 (48.7) |  | 1.00 | 1.00 | 25 (21.0) |
| **Taking diabetes drugs** | | |  |  |  |  |  |  |
| Yes | 18 | 0.70 (0.28-1.71)  0.47 | 0.71 (0.22-2.37)  0.58 | 8 (44.4) |  | 1.23 (0.46-3.31)  0.78 | 0.99 (0.23-4.15)  0.98 | 5 (27.8) |
| No | 246 | 1.00 | 1.00 | 133 (54.1) |  | 1.00 | 1.00 | 58 (23.6) |
| **Taking hypnotics** | |  |  |  |  |  |  |  |
| Yes | 46 | 1.04 (0.61-1.76)  1.00 | 1.32 (0.65-2.68)  0.45 | 25 (54.3) |  | 1.13 (0.62-2.04)  0.71 | 0.97（0.42-2.23）  0.94 | 12 (26.1) |
| No | 218 | 1.00 | 1.00 | 116 (53.2) |  | 1.00 | 1.00 | 51 (23.4) |
| **Relocation from home** | | |  |  |  |  |  |  |
| Yes | 104 | 1.45 (1.06-1.99)  0.02 | 2.17 (1.25-3.78)  <0.01 | 65 (62.5) |  | 1.01 (0.71-1.43)  1.00 | 0.96 (0.49-1.87)  0.91 | 25 (24.0) |
| No | 160 | 1.00 | 1.00 | 76 (47.5) |  | 1.00 | 1.00 | 38 (23.8) |

*Note.* COR = crude odds ratio from bi-variable analysis. ; AOR = adjusted odds ratio from multinomial logistic regression analysis. ; CI = confidence interval.

^a^ The referent categories are in the last row for all the explanatory variables.
